# Engaging Leadership and Psychological Safety as Moderators of the Relationship between Strain and Work Recovery: A Cross-Sectional Study of HSS Employees

**DOI:** 10.3390/healthcare11071045

**Published:** 2023-04-05

**Authors:** Kirsikka Selander, Eveliina Korkiakangas, Minna Toivanen, Kirsi Yli-Kaitala, Hilpi Kangas, Nina Nevanperä, Jaana Laitinen

**Affiliations:** 1 Finnish Institute of Occupational Health, Työterveyslaitos, 70032 Kuopio, Finland; 2 Finnish Institute of Occupational Health, Työterveyslaitos, 90032 Oulu, Finland; 3 Finnish Institute of Occupational Health, Työterveyslaitos, 00032 Helsinki, Finland

**Keywords:** recovery from work, health and social services, eldercare, job demands, job resources, job strain, engaging leadership, psychological safety of work community

## Abstract

Work in the health and social sector (HSS) is highly straining and therefore recovery from work needs to be promoted. Less is known on how job resources can be used to alleviate job strain and increase recovery from work. Thus, we analyzed the following: the association between job demands and work recovery; the connections of engaging leadership and psychological safety to recovery from work; and the moderating effects of engaging leadership and psychological safety on the relationship between strain and recovery from work. This cross-sectional study of 18,155 HSS and 4347 eldercare employees in 2020 using linear regression analysis showed that job strain (*p* < 0.001) and moral distress (*p* < 0.001) were associated with decreased recovery from work. Engaging leadership (*p* < 0.001) and psychologically safe work community (*p* < 0.001) enhanced recovery from work independently. Engaging leadership mitigated the harmful effect of job strain (*p* < 0.01) and moral distress (*p* < 0.05), and psychological safety mitigated the effect of job strain (*p* < 0.001), but not moral distress (*p* > 0.05). Thus, it is important to reduce job strain so that employees recover from work. Further job resources such as engaging leadership and psychological safety are important in themselves as they support recovery from work and employees’ well-being, but also as they alleviate job demands.

## 1. Introduction

Health and social services in Finland as well as in many other countries face challenges due to a simultaneous lack of work force and an increasing number of elderly patients. The shortage of employees increases individual workers’ workload, which together with other work stressors, such as low job control and moral distress, may cause increased stress, decreased work ability and poor health, especially if the stressors accumulate [1,2]. Employees in eldercare experience job strain and moral distress more often than HSS employees in general, which further lowers their work ability. Most harmful for work ability is if job strain and moral distress accumulate with other psychosocial stressors. Thus, HSS organizations should pay more attention to lowering moral distress and workload [1]. 

Moral distress derives from situations where the employee is unsure about the right way to act, or from situations where the employee has to act against set guidelines and rules, or against one’s own values and ethical principles [3,4]. Many employees in HSS and elder care do shift work, which is a further risk factor for health and work ability [5,6]. Sustaining the work careers of HSS workers requires preventing the harmful effects of the accumulation of job strain and moral distress on their health and work ability. One way to achieve this is to promote recovery from work [7,8].

Recovery from work is a process in which the psychophysiological systems that are activated during work return to their baseline level, and during which resources are replenished [9,10]. Earlier research has mainly focused on the individual ways to recover from work, such as recovery enhancing activities (physical exercise and hobbies) and recovery experiences [11,12]. Organizational level studies have focused on ergonomic shift scheduling [13,14] and recovery during breaks [15,16]. Moreover, leisure time recovery between work shifts [17] and during weekends [18], as well as vacations [19], have been investigated. 

Theoretically, recovery from work can be linked to the processes of “health impairment” and “motivational” process in the Job Demands–Resources model [20]. In the health impairment process, job demands, such as excessive workload, require constant effort that exhaust employees’ mental and physical resources. This may lead to the depletion of energy and thus increase the need for recovery. In the “motivational process”, job resources such as job autonomy and feedback from supervisors help employees in achieving work goals. This prevents the exhaustion of employees’ resources [21,22,23].

The JD-R model has been previously used in the context of recovery studies, for example by testing the role of recovery experiences (psychological detachment from work, relaxation, mastery, and control during leisure time) as mediating factor between work characteristics and well-being/health (e.g., [20]). Findings have provided support to health impairment and motivational processes as well as a negative relationship with job demands and a positive association with job resources [9,24]. Yet, the role of resources has received less attention in the recovery process than demands and there is a gap related to moderation effects [9], such as the role of engaging leadership and a psychologically safe work community, on recovery from work. 

In this article, our objective was to analyze factors associated with recovery from work. Specifically looking at job demands, we concentrated on job strain and moral distress, which are emphasized especially in eldercare [1]. Thus, our first hypothesis was (H1) “*Job strain and moral distress are negatively associated with recovery from work*”. Because it is difficult to decrease work strain rapidly due to population aging and a lack of workforce, there is a need to understand how job resources can be used to alleviate job demands and enhance recovery from work. Therefore, our second aim was to analyze whether leadership and work community can be used in alleviating job strain and moral distress and whether they are associated with recovery from work. 

### Potential Job Resouces

Leadership conceptualized differently (e.g., transformational leadership, Leader-Member Exchance (LMX), supervisor support) has been most often used as job resource in the JD-R model (e.g., [25,26]). Wilmar Schaufeli [27], however, was the first who suggested that leadership should not be considered barely as a job resource (or job demand) as leaders have important role in balancing their followers job demands and resources. First, leaders can directly impact job demands (e.g., amount of work) and job resources (e.g., worktime autonomy). Secondly, even though they may not be able to reduce the workload totally, they can provide more resources and thus alleviate the negative association of job demands on recovery from work [26]. To analyze leadership, Wilmar Schaufeli [27] developed the concept of “engaging leadership” based on self-determination theory (SDT) [28]. 

Engaging leadership enables the fulfillment of basic psychological needs and creates a resourceful work context in which the employees can thrive [27,29]. Engaging leadership is seen as behavior of the leader that aims to facilitate (empower), strengthen, connect, and inspire followers, and through that, for example, increase their work engagement [27,30]. To our knowledge, the link between engaging leadership and recovery from work is still vague and should be studied in more depth. In this study, we hypothesized (H2) whether “*engaging leadership is associated with recovery from work*”.

Even though the direct link is mostly unexplored, leadership and other stable work-related resources are shown to be essential for reducing and preventing job strain [31]. Some previous research has, further, found that servant leadership, which can be seen as a related theory to engaging leadership, is negatively connected to strain [32], which could lead to a lowered need for recovery from work. Based on this, we hypothesized that (H3) “*engaging leadership moderates the association between job demands and recovery from work*”.

In addition to leadership, the work community can also act as job resource, but as with leadership it is rarely analyzed as a balancing force between job demands and recovery from work. In this article we concentrate on psychological safety, which refers to a shared belief that the group is safe for interpersonal risk taking, such as admitting mistakes or speaking up about concerns [33]. In a psychologically safe work community, ideas are shared, and problems are brought up openly [33,34]. It can be assumed that high psychological safety supports psychological recovery from work when problems and ethically challenging situations can be dealt with openly at the workplace. This helps psychological detachment from work [35]. In previous studies, psychological safety has been found to be connected to, for example, social sharing, organizational and work commitment, job satisfaction and, in health care, also patient safety and curbing employee burn out [36,37,38], but to our knowledge no studies exist in relation to recovery from work. In this study we hypothesized (H4) whether “*psychological safety is associated with recovery from work*”. 

Furthermore, open discussion can help to identify employees’ excessive workloads, facilitate the division and equalization of work duties, and ultimately reduce workload. Psychological safety can thus moderate the association between job demands and work recovery. However, reliable research-based information on these connections is not available. In this article we hypothesized (H5) that “*psychological safety moderates the association between job demands and recovery from work*”.

## 2. Materials and Methods

In Finland, as in other Nordic countries, every resident is entitled to adequate social, health and medical services, which are provided by public institutions. Private social and health care organizations complement public services. Because of central role of public institutions data collection concentrated on nine Finnish public sector organizations. Data were collected between October and November 2020. All employees who were actively working in these organizations (excluding those in parental, sick or study leave) received a link to the electronic survey. Of the invited employees 24,459 responded (response rate 67%) and 92% gave an informed consent to use the data for research purposes (N = 22,502). Those who consented to research were further classified into general HSS (N = 18,155) and eldercare (N = 4347) employees based on work unit names. Eldercare included work units in which work involves close contact with the elderly, including immediate superiors. Administrative work, top management and all other work units were included in the general HSS.

### 2.1. Measures

Recovery from work was measured with single-item recovery scale developed by Kinnunen and Feldt [39]. Respondents were asked to assess whether they recover from the strain caused by the workday before the next day on a scale from 0 (not at all) to 10 (completely).

Job strain was measured with four items from the Job Content Questionnaire [40]. Job demands (e.g., I am required to do an unreasonable amount of work) and job control (e.g., I have lot of say in my own work) both included two questions with a response scale from 1 = strongly agree to 5 = strongly disagree. Job strain was calculated by subtracting the mean of job control from job demands [41].

Moral distress follows Nash’s theory [42] of moral distress. It was measured with a mean sum variable (Cronbach alpha = 0.78) consisting of two questions (e.g., “How often do you have to act against rules and norms?) with a five-point Likert-type scale, 1 = never to 5 = daily. 

Engaging leadership follows Wilmar Schaufeli’s [27] conceptualization. It is a mean sum variable (Cronbach alpha = 0.95) including nine statements (e.g., my supervisor encourages team members to develop their talents as much as possible) with response options from 1 (totally disagree) to 5 (totally agree).

Psychological safety is a part of the Team Climate Inventory (TCI) scale developed by Anderson and West [43]. In this study we used seven questions (e.g., everyone feels understood and accepted) with response options from 1 (totally disagree) to 5 (totally agree). In the analysis they are used as a mean sum variable (Cronbach alpha = 0.91). 

The covariates used in this study include gender (female/male), age, supervisory position (yes/no), shift work (yes/no), poor perceived health (health rated lower than good on a 5-item scale, [44] and occupation (administration and clerical, nutrition and cleaning, practical nurse, nurse, social workers and social counselors, others).

### 2.2. Methods

We used hierarchical linear regression analysis to test associations, including moderation effects. In order to reduce multicollinearity in the analysis, all variables were centered before the analysis by subtracting the mean from each variable [45]. 

## 3. Results

The demographics of the data are presented in Table 1. Most of the respondents were females, aged 35 to 54 years and work in non-supervisory positions. In the eldercare the employees were a bit older, and the proportion of females and non-supervisors was even higher than in general HSS. Shift work was also more common, and employees perceived their health poor more often than employees in other parts of HSS. In the eldercare most of the employees worked as practical nurses, whereas elsewhere in HSS employees were more evenly distributed across different occupations. 

Moreover, recovery from work among eldercare employees (mean = 5.86, 95% CI 5.79–5.94) was at a lower level than among other HSS employees (mean = 6.48, 95% CI 6.44–6.51). Recovery from work was also at a lower level among females, non-supervisors, employees in shift work, employees with poor health or those who work as practical nurses, which are characteristics more common in the eldercare.

### Associations between Work Recovery, Job Demands and Job Resources

The results of the linear regression analysis are presented in Table 2. Eldercare and control variables were entered in the regression model in steps 1 and 2. As can be seen from Table 2, recovery from work among eldercare employees was at a lower level even after adjusting for demographics, but the difference disappeared in the third step. Differences in other demographic variables, however, mostly remained. Males, older employees, supervisors, employees in regular work and with good health recovered from work better than females, younger employees, non-supervisors, employees in shift work or with poor health. Of the occupations, recovery from work was at a lower level among practical nurses and nurses, and in the later steps also among social workers and social counselors. Administration and clerical workers, in turn, recovered from work better than employees in other HSS occupations.

Job strain and moral distress were included in the third step, and job resources—engaging leadership and psychological safety—in the fourth step. As Table 2 shows, job strain and moral distress were negatively associated with recovery from work, whereas job resources had a positive association with it. This gave support for hypothesis 1, 2 and 5. Changes in the explanation power and the sizes of regression coefficients further showed that job demands had a stronger association with recovery from work than job resources. Adding job resources increased explanation power only by one percentage point. 

**Figure 1:**
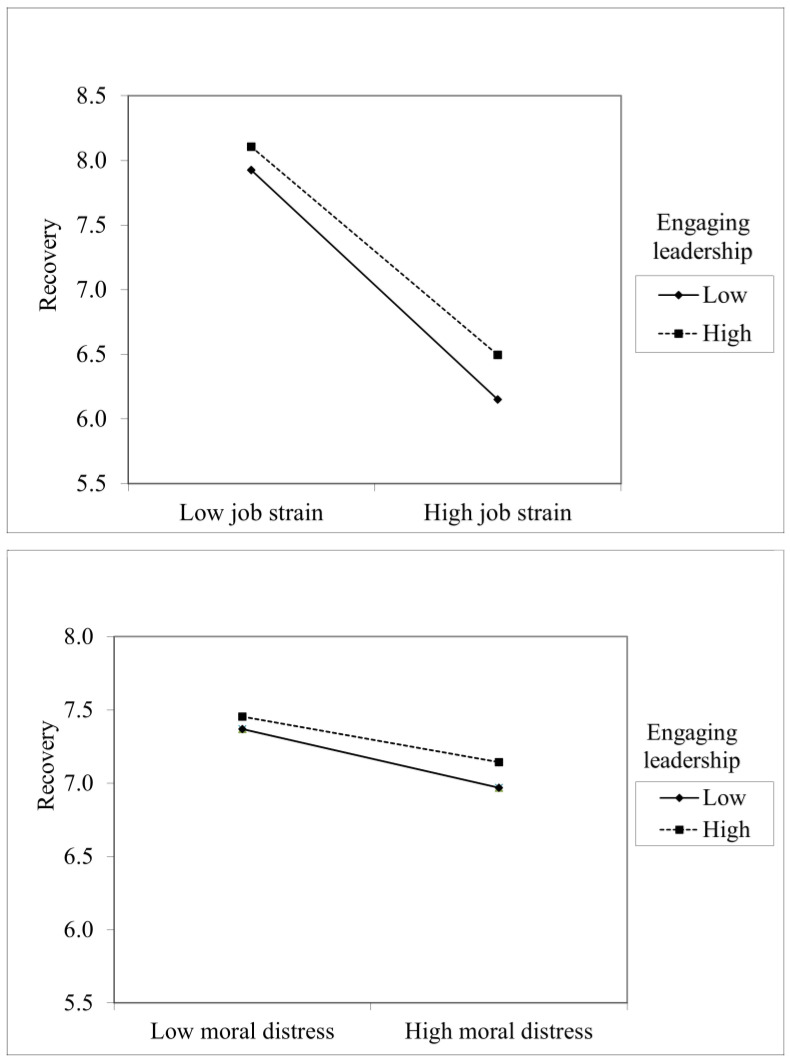
Engaging leadership moderates the effects of job strain and moral distress.

**Figure 2:**
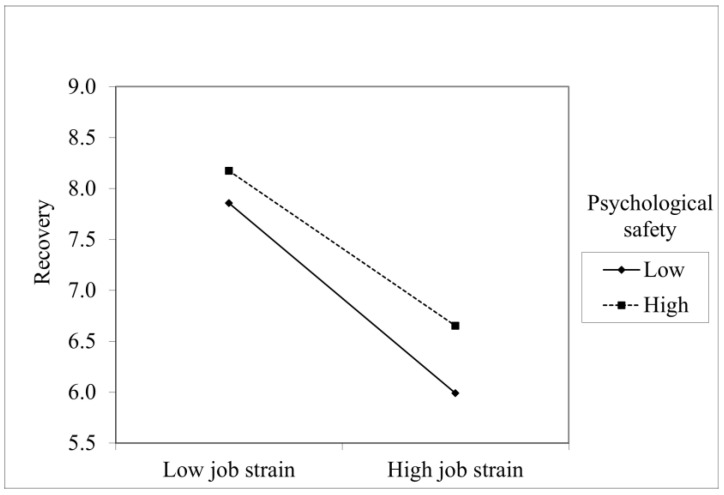
Psychological safety moderates the effects of job strain.

Interaction effects were entered in the final step. Engaging leadership had interaction effect with job strain and moral distress giving support for hypothesis 3. Psychological safety had interaction effect only with job strain Thus, hypothesis 5 was only partially supported. The effects of interaction, however, were relatively weak (see Table 2). Figure 1 and Figure 2 present statistically significant interaction effects. 

## 4. Discussion

This study on 22,500 HSS employees showed that job strain and moral distress were associated with decreased recovery from work, thus giving support for hypothesis 1. Job resources, such as engaging leadership and a psychologically safe work community, enhanced recovery from work independently, and additionally, they also mitigated the harmful effect of job strain on recovery from work somewhat, but not totally. This gave support for hypothesis 2–4 and partially for hypothesis 5. Psychological safety moderated only the association between job strain and recovery from work, not between moral distress and work recovery.

Job demands had the strongest association with recovery from work, which is in the line with the JD-R model’s assumptions of the health impairment process [20,21,22,23]. Reducing job strain and moral distress are important but challenging tasks in HSS and in eldercare to improve the attractiveness of these sectors [46]. However, there are only a few interventions that have developed work and work processes to reduce job demands or moral distress, or to enhance job control [47,48]. This is the case despite evidence that job strain is an important risk factor for mental disorders [49] and, alone or in combination with other psychosocial factors, even for work disability [1,2]. Promoting recovery from work is key to preventing the development of harmful effects of accumulating and longitudinal stress on health and work ability [1], and is important in the attempt to enhance HSS careers. While waiting for effective interventions to develop healthy work, organizations should implement norms and practices that encourage employees to recover from work during and outside the work shifts. This includes an organizational culture that supports employees having regular breaks during workdays [8] and regulating contacting employees outside the work shift that may cut recovery experiences [9]. Organizations may also support an atmosphere that encourages employees to keep up healthy lifestyles that enhance recovery from work, such as a decent amount of sleep and exercising [50,51,52,53].

Our results showed that both engaging leadership and psychological safety of the work community had a weak positive connection to recovery from work and can, to some extent, alleviate the negative association between job strain and work recovery. Thus, engaging leadership and psychological safety are job resources and increasing them supports recovery from work. Moreover, this study brought into discussion the role of leadership and psychological safety within the context of job resources and demands. In line with the findings of Schaufeli [27] our study implies that engaging leadership and psychological safety of work community are not valuable only as such, but leadership and work community play important roles in alleviating the negative association between job demands and recovery from work. Currently, most nurse leaders spend most of their working time locating resources and managing day-to-day nursing activities and their development. To promote sustainable working careers, the role of the nurse leaders should be developed so that it allows for promoting recovery from work and well-being at the workplace. Engaging leadership enhanced recovery from work, which to our knowledge is a novel finding. It further alleviated job strain, which supports previous findings [9,32]. Stress, including e.g., job strain, are contextual elements within work, and a result of frustration of basic psychological needs [54]. Engaging leadership is seen as a behavior of the leader, which enables the satisfaction of employees’ basic psychological needs [27]. Leaders also play a crucial role in fostering employees’ psychological safety [36]. Characteristics such as open communication, trust and support help to develop an atmosphere of psychological safety [55]. These are also features that are associated with engaging and servant leadership styles; and servant leadership has indeed been shown to enhance employees’ psychological safety [56,57]. In addition, psychological safety has been found to act as a mediating factor between leadership and well-being [38]. So, the associations we examined may be influenced by the strong connection between leadership and psychological safety. In the future, the mediating effect of psychological safety between leadership and recovery from work should be analyzed in more detail.

The main strength of this study is the novel information that it provides on engaging leadership and psychological safety as ways to mitigate HSS employees’ job strain and enhance recovery from work. Our dataset covered a large number of Finnish HSS care employees with a reasonable response rate (67%). It also included a large number of eldercare respondents, who to our knowledge, are less studied as a target group in research related to the promotion of work ability and recovery from work. 

Furthermore, the use of an extensive survey of HSS employees is a strength, but also a limitation that should be highlighted to the reader. As the survey covered a wide range of questions, we had to use abbreviated versions of measurements, so that response activity would not deteriorate too much. For example, the measurement scale of psychological safety was a shortened version of the Team Climate Inventory scale. The questions used only partially reach the concept of psychological safety. A more precise and multidimensional measure of psychological safety may have brought up stronger effects. Thus, future analysis with more precise measurements is needed. Cross-sectional study design limits us from making conclusions about causality. 

This study produces hypotheses and practical implications, although it also had limitations, as discussed. Despite them, our results can be used in formulating two hypotheses for workplace action strategies to be tested in intervention studies or in pseudo trials. The first strategy is to reduce job strain and moral distress, as when there is less strain at work there is less need for recovery from work. The second strategy is to enhance job resources related to leadership and work community. HSS work communities need to increase the psychological safety of the work community and further develop leadership toward engaging leadership that promotes recovery from work. This is important in order to promote good work ability and sustainable working careers among HSS workers and to tackle the increasing labor shortage in the HSS workforce, especially in eldercare. 

## 5. Conclusions

It is important to reduce job strain in the HSS sector so that employees recover from work, and so that prolonged, accumulated work strain does not cause health problems and reduce work ability. Increasing job resources is important, and engaging leadership and the psychological safety of the work community can support recovery from work and employees’ well-being, although they do not remove the negative association between job strain and recovery from work. Thus, leadership should focus on the activities to promote recovery from work at the workplace and work community. These are possible actions in tackling the labor shortage in the HSS sector.

## Figures and Tables

**Table 1:** Demographics and recovery from work in different employee groups. The table presents sample sizes, mean and 95 % confidence intervals (CI) for recovery from work.

	n (%)				Recovery from Work, Mean (95%CI)	
	All HSS Employees	Eldercare	Other HSS Employees	All HSS Employees	Eldercare	Other HSS Employees
Gender						
Males	2950 (13%)	194 (4%)	2756 (15%)	6.91 (6.82–6.99)	6.26 (5.91–6.62)	6.95 (6.86–7.04)
Females	19,552 (87%)	4153 (96%)	15,399 (85%)	6.28 (6.24–6.31)	5.84 (5.77–5.92)	6.39 (6.36–6.43)
Age						
Less than 35	5135 (23%)	925 (21%)	4210 (23%)	6.07 (6.00–6.13)	5.41 (5.25–5.58)	6.21 (6.14–6.28)
35–54	11,722 (52%)	2137 (49%)	9585 (53%)	6.38 (6.34–6.43)	5.90 (5.79–6.00)	6.49 (6.45–6.54)
55+	5645 (25%)	1285 (30%)	4360 (24%)	6.57 (6.51–6.54)	6.13 (5.99–6.26)	6.70 (6.63–6.77)
Supervisory position						
Yes	20,653 (8%)	177 (4%)	1590 (9%)	7.02 (6.91–7.14)	6.85 (6.32–7.38)	7.03 (6.91–7.15)
No	1767 (92%)	4156 (96%)	16,497 (91%)	6.26 (6.23–6.29)	5.80 (5.72–5.88)	6.38 (6.35–6.42)
Shift work						
Yes	10,813 (48%)	3371 (78%)	7442 (41%)	5.80 (5.75–5.85)	5.60 (5.52–5.69)	5.89 (5.83–5.95)
No	11,639 (52%)	966 (22%)	10,673 (59%)	6.84 (6.79–6.88)	6.71 (6.55–6.87)	6.85 (6.80–6.89)
Perceived health						
Poor	5434 (24%)	1327 (31%)	4107 (23%)	4.95 (4.88–5.01)	4.58 (4.45–4.71)	5.06 (4.99–5.14)
Good	17,022 (76%)	3011 (69%)	14,011 (73%)	6.81 (6.78–6.84)	6.43 (6.34–6.51)	6.89 (6.86–6.93)
Occupations						
Administration and clerical	2794 (13%)	119 (3%)	2675 (16%)	7.06 (6.97–7.14)	6.97 (6.60–7.35)	7.06 (6.98–7.15)
Nutrition and cleaning	725 (4%)	86 (2%)	639 (4%)	6.29 (6.11–6.47)	6.60 (6.08–7.13)	6.25 (6.05–6.44)
Practical nurses	4816 (23%)	3015 (72%)	1801 (11%)	5.92 (5.85–5.99)	5.66 (5.56–5.75)	6.36 (6.25–6.47)
Nurses	7068 (34%)	689 (16%)	6379 (38%)	5.94 (5.88–5.99)	5.91 (5.72–6.09)	5.94 (5.88–6.00)
Social workers and social counselors	2081 (10%)	173 (4%)	1908 (12%)	6.61 (6.52–6.71)	6.48 (6.13–6.83)	6.63 (6.53–6.73)
Other HSS occupations	3367 (16%)	118 (3%)	3249 (20%)	6.85 (6.77–6.92)	6.85 (6.41–7.28)	6.85 (6.77–6.93)
N	22,247	4276	17,971	6.36 (6.33–6.39)	5.86 (5.79–5.94)	6.48 (6.44–6.51)

**Table 2:** Associations between recovery from work, job demands, and job resources based on linear regression analysis. Standardized regression coefficients and their statistical significance are presented. Job strain, ethical strain, engaging leadership and psychological safety variables were centered before analysis.

	Step1	Step2	Step3	Step4	Step5
Eldercare (ref. = other HSS employees)	−0.10 ***	−0.02 **	0.01	0.03	0.00
Gender (ref. = females)		0.07 ***	0.04 ***	0.04 ***	0.04 ***
Age		0.10 ***	0.09 ***	0.10 ***	0.10 ***
Supervisory position (ref. = yes)		−0.02 **	−0.01 *	−0.03 ***	−0.03 ***
Shift work (ref. = yes)		0.16 ***	0.10 ***	0.10 ***	0.10 ***
Perceived health (ref. = poor)		0.34 ***	0.26 ***	0.25 ***	0.26 ***
Occupation (ref. = other HSS occupations)					
Administration and clerical		0.02 *	0.02 *	0.02 *	0.02 **
Nutrition and cleaning		−0.00	−0.00	0.00	0.00
Practical nurse		−0.04 ***	−0.01	−0.01	−0.01
Nurse		−0.10 ***	−0.03 **	−0.03 **	−0.03 ***
Social workers and social counselors		−0.01	−0.02 **	−0.02 **	−0.02 ***
Job strain			−0.39 ***	−0.35 ***	−0.35 ***
Moral distress			−0.10 ***	−0.09 ***	−0.09 ***
Engaging leadership				0.05 ***	0.05 ***
Psychological safety				0.08 ***	0.08 ***
Engaging leadership*Job strain					0.02 **
Engaging leadership*Moral distress					0.02 *
Psychological safety*Job strain					0.03 ***
Psychological safety*Moral distress					0.01
F change (df1, df2)	226.25 (1, 22,077) ***	446.24 (10, 22,067) ***	2715.82 (2, 22,065) ***	195.69 (2, 22,063) ***	23.33 (4, 22,059) ***
Adj. R^2^	0.01	0.18	0.34	0.35	0.35

*** *p* < 0.001; ** *p* < 0.01; * *p* < 0.05.

## Data Availability

Data are available upon reasonable request and subject to administrative approval.
